# Metabolomic Profiling in Mouse Model of Menopause-Associated Asthma

**DOI:** 10.3390/metabo13040546

**Published:** 2023-04-11

**Authors:** William P. Pederson, Laurie M. Ellerman, Yan Jin, Haiwei Gu, Julie G. Ledford

**Affiliations:** 1 Physiological Sciences GIDP, University of Arizona, Tucson, AZ 85724, USA; wpederson@arizona.edu; 2 Department of Physiology, University of Arizona, Tucson, AZ 85724, USA; 3 Asthma and Airway Disease Research Center, Tucson, AZ 85724, USA; 4 Center for Translational Science, Florida International University, Port St. Lucie, FL 34987, USA; 5 Department of Cellular and Molecular Medicine, University of Arizona, Tucson, AZ 85724, USA

**Keywords:** metabolomics, asthma, menopause, HDM

## Abstract

Menopause-associated asthma impacts a subset of women, tends to be more severe, and is less responsive to current treatments. We recently developed a model of menopause-associated asthma using 4-Vinylcyclohexene Diepoxide (VCD) and house dust mites (HDM). The goal of this study was to uncover potential biomarkers and drivers of menopause-onset asthma by assessing serum and bronchoalveolar lavage fluid (BALF) samples from mice with and without menopause and HDM challenge by large-scale targeted metabolomics. Female mice were treated with VCD/HDM to model menopause-associated asthma, and serum and BALF samples were processed for large-scale targeted metabolomic assessment. Liquid chromatography–tandem mass spectrometry (LC-MS/MS) was used to examine metabolites of potential biological significance. We identified over 50 individual metabolites, impacting 46 metabolic pathways, in the serum and BALF that were significantly different across the four study groups. In particular, glutamate, GABA, phosphocreatine, and pyroglutamic acid, which are involved in glutamate/glutamine, glutathione, and arginine and proline metabolisms, were significantly impacted in the menopausal HDM-challenged mice. Additionally, several metabolites had significant correlations with total airway resistance including glutamic acid, histamine, uridine, cytosine, cytidine, and acetamide. Using metabolic profiling, we identified metabolites and metabolic pathways that may aid in discriminating potential biomarkers for and drivers of menopause-associated asthma.

## 1. Introduction

Asthma is a common respiratory disease, characterized by episodic, and reversible, airway obstruction, affecting nearly 8% of the US population [1]. Symptoms include wheezing, coughing, and shortness of breath, all of which contribute to an inability to breathe [2,3,4]. However, asthma is a heterogeneous disease, with prevalence and severity varying wildly across age, sex, and ethnicity, emphasizing environmental and genetic factors that contribute to disease pathogenesis [5]. This heterogeneity has led researchers to identify and characterize specific asthma endotypes based on symptoms and cellular and molecular phenotypes [5,6]. The most common of these endotypes is allergic asthma, which involves eosinophilic inflammation in the airways in response to allergenic stimuli [6]. Allergic asthma, along with several other common asthma endotypes, has been well studied, and there are a variety of treatments and therapies available. However, one subtype of asthma that remains less well defined is menopause-associated asthma [7,8].

Women experiencing menopause-associated asthma often encounter more severe symptoms, which often require hospitalization and advanced treatment beyond the standard of care [9]. As such, late-onset asthma in menopausal women often goes uncontrolled, since standard asthma treatments are relatively ineffective [7]. Recently, our lab developed a mouse model of menopause-associated asthma in order to study the mechanisms driving disease onset associated with menopause and identify differences from non-menopause onset asthma [8]. Utilizing the 4-Vinylcyclohexene Diepoxide (VCD) mouse model of menopause, in combination with the house dust mite (HDM) mouse model of asthma, we were able to demonstrate significantly increased airway hyper-responsiveness and non-Th2 airway inflammation in menopausal, HDM-challenged mice [8]. To the best of our knowledge, this is the first animal model to mimic clinical phenotypes observed in women with menopause-associated asthma.

Despite the success of the VCD/HDM model in replicating the severe airway function and non-Th2 inflammation seen in women, the underlying molecular mechanisms remain unknown. It is crucial to have a comprehensive understanding of cellular and molecular mechanisms of asthma in order to identify biomarkers unique to specific endotypes and to discover novel targets for new therapies. In recent years, metabolomics, or quantitative analysis of small molecules in biological samples, has become a powerful tool for identifying mechanisms of complex diseases, including asthma [10,11,12]. Metabolomic studies performed on human samples, including blood, lung tissue, sputum, urine, and exhaled breath condensates, have reported a wide range of metabolites and metabolic pathways that distinguish asthma subtypes based on age, asthma severity, obesity, and other factors [12,13]. Some of the most commonly reported metabolic pathways affected by asthma include lipid metabolism, amino acid metabolism, and oxidative stress [11]. While there is an abundance of metabolomic data describing potential mechanisms for asthma, these studies also highlight the severe gap in our understanding of the complexity of the signaling pathways at play. Additional metabolomic studies and comprehensive review of reported metabolic pathways is necessary to develop a stronger understanding of asthma endotypes and their respective cellular and molecular mechanisms.

In this study, we performed large-scale targeted metabolomics on samples from mice from two compartments, bronchoalveolar lavage fluid (BALF) and serum, across four experimental conditions: control, HDM challenged, menopausal non-HDM challenged, and menopausal HDM challenged. These findings contribute to the current metabolomic profiles being developed for asthma endotypes in humans and shed light on potential biomarkers that could be used to discriminate menopause-associated asthma from non-menopause asthma, in addition to providing novel therapeutic targets for a group of patients with limited treatment options.

## 2. Materials and Methods

**Figure 1:**
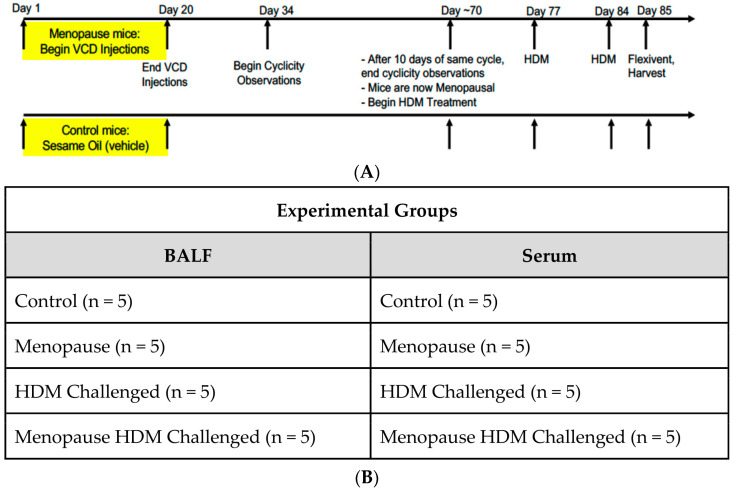
Experimental setup. (**A**) Timeline of the VCD/HDM model denoting times of VCD dosing, cyclicity checking, HDM dosing, Flexivent and tissue harvest. (**B**) Table denoting the four experimental groups, as well as n values per group.

Mouse Models: All experiments utilizing animal models were performed in accordance with University of Arizona Institutional Animal Care and Use Committee-approved animal protocols. Wild-Type (WT) female mice on a C57BL/6J background were ordered from Jackson Laboratories. Mice were age-matched (7 weeks) at the time of delivery, and experiments began when age reached 8 weeks. As shown in Figure 1A, intraperitoneal injections of the VCD compound (160 mg/kg, Sigma, St. Louis, MO, USA, #94956) in sesame oil (Sigma, #S3547), or sesame oil alone as vehicle control, were performed daily for twenty consecutive days [8]. Two weeks following the final IP injection, cyclicity was performed daily via a vaginal lavage, the cytology of which was monitored daily to determine the onset of menopause (ovarian failure), defined as 10 consecutive days of diestrus. Sensitization and challenge involved intranasal administration of HDM (Greer Laboratories, Lenoir, NC, USA, #NC1468783), or saline vehicle control, once a week for a total of three weeks. Mice that received HDM were given 50 μL of solution at a concentration of 2 mg/mL [8].

Sample Collection: Twenty-four hours after the third HDM challenge, mice were weighed, and lung function tests were performed on the flexiVent system (SciReq, Montreal, QC, Canada) [8]. Bronchoalveolar Lavage Fluid (BALF) was obtained by gently flushing the lungs with 1.5 mL of lavage fluid (PBS, 0.1 mM EDTA) three times. Mice were dissected, and blood was collected via cardiac puncture. Blood samples were centrifuged to separate the serum, and serum was transferred to a new tube. Cells suspended in solution in the BALF were separated via centrifugation, and the cell-free BALF was aliquoted into new tubes. BALF and serum samples were stored at −80 °C from the 4 experimental groups as detailed in Figure 1B until further analysis.

Metabolomics Analysis: Before metabolomic analysis was performed, protein precipitation and metabolite extraction were performed on all samples. To begin, 50 μL of either BALF and serum sample were transferred to a 2 mL Eppendorf tube after being thawed overnight at 4 °C. Then, 500 μL MeOH and 50 μL internal standard solution (containing 1810.5 μM 13C3-lactate and 142 μM 13C5-glutamic acid) was added to each sample, followed by vortexing for 10 s, storage at −20 °C for 30 min, and finally centrifugation at 14,000 RPM for 10 min at 4 °C. The supernatants, typically around 450 μL, were then transferred to a new Eppendorf tube, dried using a CentriVap Concentrator (Labconco, Fort Scott, KS, USA), and reconstituted in 150 μL of 40% PBS/60% ACN. A quality control (QC) sample was created by pooling all plasma samples together. We used a targeted LC-MS/MS method for metabolomic assessment that was modeled after methods used regularly in a large number of studies [14,15,16,17,18,19,20]. All LC-MS/MS experiments were performed utilizing the Agilent 1290 UPLC-6495 QQQ-MS (Santa Clara, CA, USA) system. Each sample was analyzed using negative ionization mode (using 10 μL of total sample) and positive ionization mode (using 4 μL of total sample). Hydrophilic interaction chromatography (HILIC) mode on a Waters XBridge BEH Amide column (150 × 2.1 mm, 2.5 µm particle size, Waters Corporation, Milford, MA, USA) was used for both chromatographic separations. The auto-sampler temperature was kept at 4 °C, the column compartment was set at 40 °C, and the flow rate was 0.3 mL/min. Solvents A (10 mM ammonium acetate, 10 mM ammonium hydroxide in 95% H_2_O/5% ACN) and B (10 mM ammonium acetate, 10 mM ammonium hydroxide in 95% ACN/5% H_2_O) made up the mobile phase. The percentage of Solvent B decreased to 40% at *t* = 11 min after the initial 1 min isocratic elution of 90% B. The composition of Solvent B was then maintained at 40% for 4 min (*t* = 15 min), before gradually increasing to 90% to prepare for the next injection. An electrospray ionization (ESI) source was equipped to the mass spectrometer and targeted data acquisition was performed in multiple-reaction-monitoring (MRM) mode. Agilent Masshunter Workstation software (version 10.1, Santa Clara, CA, USA) controller the entire LC-MS system. Agilent MassHunter Quantitative Data Analysis (Santa Clara, CA, USA) was used to integrate the extracted MRM peaks [14,15,16,17,18,19,20].

Statistical Analysis: Statistical analysis of previously published airway function data [8] was account for variations of recorded data on separate experimental days, and statistical significance was assessed using unpaired *t*-tests, according to previous publications [8,21]. Analysis of metabolites and pathway analyses were performed using MetaboAnalyst 5.0 software. Metabolite data were normalized using log transformation, and statistical analysis was performed using an ANOVA, for comparison of all four groups together, or an unpaired *t*-test, for comparison of only two groups. For analysis using ANOVA, significance was achieved with *p* ≤ 0.05, using Tukey’s Honest Significant Difference. For analysis using unpaired *t*-test, significance was achieved with *p* ≤ 0.05, and a fold change threshold of 1.5. Pathway analysis was performed comparing each experimental group to one another. Partial least squares-discriminant analysis (PLS-DA) was performed comparing all four experimental groups.

## 3. Results

### 3.1. Impact of Menopause on Metabolomic Profile

As previously published, the HDM and menopause HDM experimental groups had a robust eosinophilic response compared to their saline controls but were not different from one another; both having >70% eosinophils and averaging ~6 × 10^5^ Eos/mL BALF [8]. The menopause HDM group had approximately twice the number and percentage of neutrophils in BALF as compared to the HDM only group [8]; however, neutrophils were much lower compared to the eosinophils, as was expected of the HDM model of allergic airways disease in mice.

By comparing the menopausal group to the controls, we observed how menopause affects metabolomic changes in the BALF and serum. There were no statistically significant upregulated metabolites in the BALF of menopausal mice compared to control, but 4-aminobutyric acid (GABA) showed an increasing trend (Table 1). 

**Figure 2:**
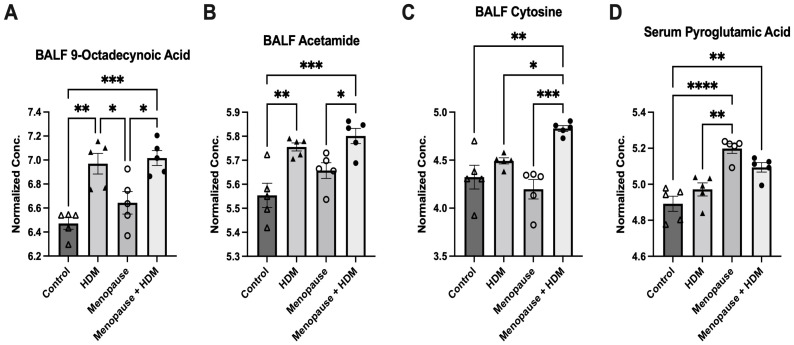
Significantly different metabolites across the four experimental groups by one-way ANOVA with multiple comparisons. (**A**) 9-Octadecynoic acid in BALF; (**B**) acetamide in BALF; (**C**) cytosine in BALF; (**D**) pyroglutamic acid in serum. * *p* ≤ 0.05, ** *p* ≤ 0.005, *** *p* ≤ 0.0005, **** *p* ≤ 0.00005.

Menopausal mice had a significant upregulation of several metabolites in the serum, including pyroglutamic acid (Figure 2D), glucosamine, 3-hydroxyisovaleric acid, myristic acid, nicotinuric acid, 4-pyrodoxic acid, oxoglutaric acid, 2-methylglutaric acid and indole 3-acetic acid compared to non-menopausal mice (Table 2).

Conversely, menopause resulted in a downregulation of several metabolites in the BALF, including TMAO, 2-hydroxybenzoic acid (salicylic acid), threonine, homoserine, hippuric acid, hydroxyproline, 5-aminolevulinic acid (Table 1). Interestingly, menopause had an even greater impact on the metabolomic profile in the serum. Menopause resulted in a significant downregulation of many metabolites in the serum, including 2-hydroxybenzoic acid (salicylic acid), oxidized glutathione, mucic acid, 5-aminolevulinic acid, acetylcholine, ribose, methyl guanidine, xylose, hydroxyproline, carnosine, uridine, and GA3P (Table 2).

**Figure 3:**
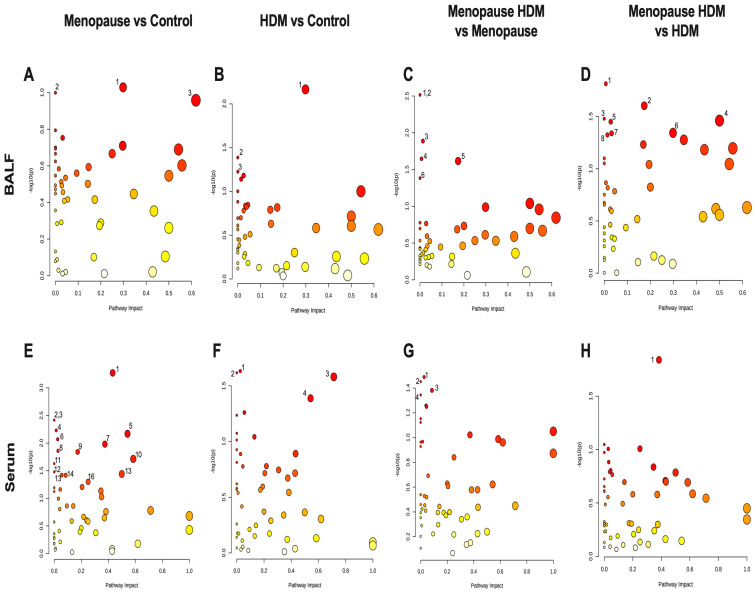
Pathway analysis plots for group comparison in BALF (**A**–**D**) and serum (**E**,**F**). (**A**,**E**) Menopause vs. Control; (**B**,**F**) HDM vs. Control, (**C**,**G**) Menopause HDM vs. Menopause; (**D**,**H**) Menopause HDM vs. HDM. The log *p*-values of the significantly different metabolites are plotted within a given metabolic pathway by the impact score that those metabolites have on the pathway. The size of the circle correlates to the number of significantly different metabolites detected for that pathway. Numbers next to circles correspond to numbers next to pathways listed in Table 3 and Table 4.

**Figure 4:**
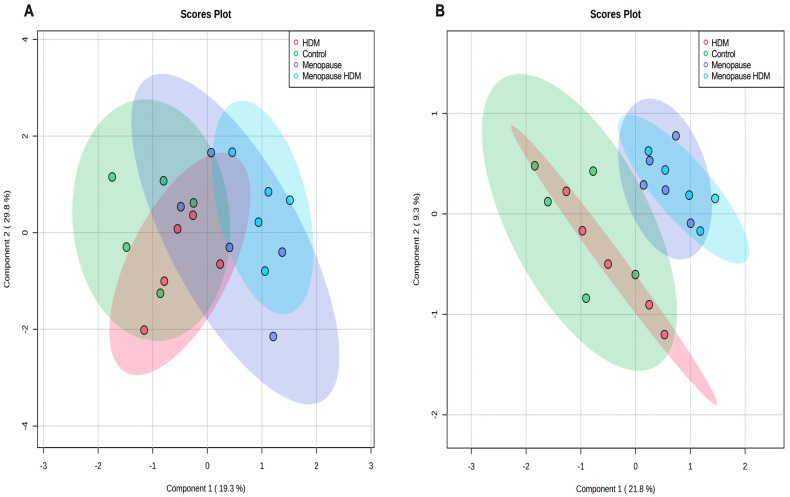
PLS-DA analysis of control, HDM-challenged, menopausal, and menopausal HDM-challenged mice. Score plots of BALF (**A**) and serum (**B**) using all significant metabolites.

Pathway analysis was performed, which groups individual metabolites into known metabolic pathways. In BALF, menopause did not significantly impact any metabolic pathways in the KEGG database (Figure 3A, Table 3). However, in serum, menopause significantly impacted 16 different known metabolic pathways, the most significantly affected being glutathione metabolism, glycine, serine, and threonine metabolism, pentose and glucuronate interconversions, pentose phosphate pathway, alanine, aspartate, and glutamate metabolism, d-glutamine and d-glutamate metabolism, and tryptophan metabolism (Figure 3E, Table 4). PLS-DA analysis of the BALF metabolites in all groups showed some overlap between menopausal and control groups, although discrimination of these two groups can be observed (Figure 4A). PLS-DA analysis of the serum metabolites shows very distinct discrimination between menopausal mice and control mice (Figure 4B).

### 3.2. Impact of HDM on Metabolomic Profile

Comparing the HDM-challenged group to the non-HDM-challenged controls, we assessed how HDM, our asthma model, affected metabolomic changes in the BALF and serum. Asthma resulted in a significant downregulation of several metabolites in the BALF, including fructose, ribose, and TMAO (Table 1), while metabolites 9-octadecynoic acid (Figure 2A), acetamide (Figure 2B), and glycocyamine were significantly upregulated in the asthma group (Table 1). When assessing the serum samples, asthma led to significant downregulation of metabolites 3-methyladipic acid, methyl-D-mannopyranoside, histamine, and carnosine (Table 2). Using a pathway analysis, we discovered that asthma significantly impacted arginine and proline metabolism, amino sugar and nucleotide sugar metabolism, and glycolysis gluconeogeneis pathways in the BALF (Figure 3B, Table 3). Additionally, asthma significantly affected the glycerophospholipid metabolism, selenocompound metabolism, histidine metabolism, and the glycine, serine, and threonine metabolism pathways in the serum (Figure 3F, Table 4). Asthma and control groups overlap entirely in the PLS-DA analysis of serum metabolites (Figure 4A). However, these groups do begin to separate when PLS-DA is performed on BALF (Figure 4B).

### 3.3. Impact of HDM in Menopausal Mice on Metabolomic Profile

By comparing the menopausal, HDM-challenged group to the menopausal control group, we assessed the impact of HDM challenge in menopausal mice. In menopausal mice, HDM challenge resulted in a significant upregulation of many metabolites in the BALF, including cytosine (Figure 2C), glycocyamine, 9-octadecynoic acid (Figure 2A), d-ribose 5-phosphate, cytidine, and phenylpyruvic acid (Table 1). In the serum, HDM challenge in menopausal mice only resulted in a significant upregulation of two metabolites: uridine and acetylcholine (Table 2). Using pathway analysis, HDM challenge in menopausal mice significantly impacted fatty acid elongation and degradation metabolism, purine metabolism, pyrimidine metabolism, pentose phosphate metabolism, and biosynthesis of unsaturated fatty acids in the BALF (Figure 3C, Table 3). In the serum, we saw significant impacts on glycerophospholipid metabolism, arachidonic metabolism, pyrimidine metabolism, and selenocompound metabolism in the menopausal HDM-challenged mice compared to the menopausal non-HDM-challenged mice (Figure 3G, Table 4). Menopausal HDM-challenged and menopause groups overlap entirely in the PLS-DA analysis of serum and BALF metabolites (Figure 4A,B).

### 3.4. Impact of Menopause in HDM-Challenged Mice on Metabolomic Profile

To distinguish menopause-associated asthma from non-menopausal asthma, we compared the metabolomic profiles of the menopausal HDM-challenged mice to the HDM-challenged control mice. In the BALF, we found significant upregulation of cytosine (Figure 2C), cytidine, glutamic acid, and d-ribose 5 phosphate in menopausal HDM-challenged mice compared to non-menopausal HDM-challenged mice (Table 1). In the serum, we observed significant upregulation of a single metabolite, phosphocreatine (Table 2). Pathway analysis of the BALF between these groups revealed significant impacts on pyrimidine metabolism, pentose phosphate metabolism, nitrogen metabolism, d-glutamine and d-glutamate metabolism, porphyrin and chlorophyll metabolism, arginine and proline metabolism, butanoate metabolism, and purine metabolism (Figure 3D, Table 3). Interestingly, a single pathway was significantly impacted in the serum, arginine and proline metabolism (Figure 3H, Table 4). PLS-DS analysis in the menopausal HDM-challenged and non-menopausal HDM-challenged group shows distinct separation of populations in the serum and BALF (Figure 4A,B).

### 3.5. Associations between Metabolites and Airway Hyper-Responsiveness

**Figure 5:**
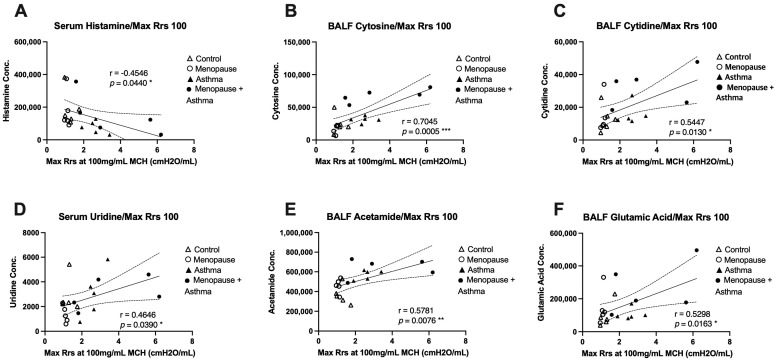
Correlation plots of metabolites with total airway resistance (Rrs). Airway resistance from the 100mg/mL methacholine dose was assessed using the Flexivent system and association analysis performed for the most significantly different metabolites detected. (**A**) Serum histamine concentration with a negative correlation to Rrs, * *p* = 0.044. (**B**) BALF cytosine concentration with a positive correlation to Rrs, *** *p* = 0.0005. (**C**) BALF cytidine with a positive correlation to Rrs, * *p* = 0.013. (**D**) Serum uridine with a positive correlation with Rrs, * *p* = 0.039. (**E**) BALF acetamide with a positive correlation to Rrs, ** *p* = 0.0076. (**F**) BALF glutamic acid with a positive correlation to Rrs, * *p* = 0.0163. All statistical significance was achieved using Pearson’s correlation coefficient.

We next wanted to assess whether any of the discovered metabolites were associated with airway function in the same mice across all experimental groups. The most significantly different metabolites were paired with the maximum total airway resistance measured at the highest dose of methacholine, which was obtained during airway function studies published previously [8]. In BALF, we found that cytosine, cytidine, acetamide, and glutamic acid were all significantly and positively correlated with total airway resistance at the 100mg/mL dose of methacholine (Figure 5B,C,E,F), indicating that when these metabolites are elevated, airway resistance is also high, and lung function is worse. In serum, we found that histamine had a significant negative correlation with total airway resistance at the 100mg/mL dose of methacholine (Figure 5A), suggesting that when histamine levels are low in the serum, airway resistance is high, and lung function is worse. Additionally, serum uridine levels had a significant positive correlation with total airway resistance (Figure 5D), indicating that when uridine is high in the serum, airway resistance is also high and lung function is worse.

## 4. Discussion

Asthma is a heterogeneous disease that consists of complex inflammatory pathways that contribute to prevalence and severity of disease. Distinct mechanistic pathways have been described to differentiate asthma endotypes and allow for the development of more precise treatments [22]. However, menopause-associated asthma has yet to be investigated in depth, and has the potential to be a distinct asthma endotype, with a unique inflammatory signature, and may require specific treatments to maintain asthma control. The lack of knowledge surrounding menopause-associated asthma demonstrates an urgent need to study and understand the molecular mechanisms driving menopause-associated asthma. In this study, we have utilized large-scale targeted metabolomics to describe the metabolomic profiles of mice undergoing the VCD/HDM model of menopause-associated asthma, with the goal of discovering novel metabolites that may serve as potential biomarkers for the identification and treatment of menopause-associated asthma. 

Metabolomics offers a powerful tool to uncover novel mechanisms of complex diseases and involves the quantitative analysis of small molecules in biological samples and their relation to pathophysiological changes in organisms [10,11,12]. Metabolomic profiling can also lead to the discovery of novel biomarkers that can help identify diseases [23]. Metabolomics has been used extensively to study asthma and airway inflammation, describing a wide variety of changes in metabolomic profiles, some of which include lipid metabolism, amino acid metabolism, energy metabolism disorders, and oxidation and reduction imbalance [11,12,13]. With this study, we aimed to describe the metabolomic profile in BALF and serum of menopause-associated asthma in the VCD/HDM model to distinguish it from non-menopausal asthma.

The metabolic pathways that are significantly impacted in the BALF of the menopausal HDM-challenged group compared to the non-menopausal HDM-challenged group share a specific metabolite: glutamic acid is a player in nitrogen, d-glutamine and d-Glutamate, porphyrin and chlorophyll, arginine and proline, and butanoate metabolic pathways. In this case, glutamic acid is the main metabolite driving the dysregulation of these metabolic pathways. glutamic acid, also known as l-glutamate has been identified in our study as significantly upregulated in the BALF of menopausal HDM-challenged mice compared to non-menopausal HDM-challenged mice. In addition, glutamate shows a positive correlation with airway resistance, indicating that higher levels of glutamate in the BALF correlate with higher levels of airway resistance. Glutamate is one of the most abundant excitatory neurotransmitters in the body, with many synapses using a variety of different glutamate receptors [24]. Additionally, glutamate is the metabolic precursor of y-aminobutyric acid, also known as GABA [25]. Previously, GABA has been shown to be secreted by pulmonary neuroendocrine cells to induce goblet cell hyperplasia in primate models [26]. GABA has also been shown to be produced by pulmonary neuroendocrine cells in murine lungs [27]. While not quite achieving statistical significance, GABA was also detected in the metabolomic assessment of BALF when comparing menopausal mice to non-menopausal mice, demonstrating a trending upregulation of the metabolite (*p* = 0.0796, FC 5.42). This suggests that menopause alone may impact neuroendocrine signaling in the lung, and in turn, affect airway epithelial cell differentiation. With increased goblet cell hyperplasia, we would also expect to see mucus hypersecretion, a key contributor to the airway obstruction seen in HDM challenged airways. However, more studies will be needed to validate these findings.

The arginine and proline metabolism pathway was the only one identified as significantly impacted in the serum analysis of the menopausal HDM-challenged mice compared to the non-menopausal HDM-challenged mice. While glutamic acid is still involved in this pathway, it was not significantly different between these two groups when individual metabolites were assessed in serum. Phosphocreatine, which was upregulated, was the only metabolite significantly different between the menopausal HDM-challenged and non-menopausal HDM-challenged groups. Interestingly, studies have shown that glutamate uptake in synaptic vesicles is not only dependent on ATP but can also be regulated by phosphocreatine [28]. While this phenomenon was demonstrated in isolated bovine brain synapses, it is possible that phosphocreatine may influence glutamate uptake in pulmonary neuroendocrine synapses as well, which could influence airway epithelial cell differentiation similar to GABA, as previously described [26]. Phosphocreatine was also upregulated in the serum of non-human primates sensitized and challenged with Ascaris suum extract and was returned to control levels with experimental treatment [29]. Having been detected in other animal models of allergic asthma, serum phosphocreatine holds potential for being a biomarker for severe asthma. However, other researchers suggest that elevated serum phosphocreatine during airway exacerbations may simply be the product of the increased work of the respiratory muscles [30]. 

Our previous work suggests that non-Th2 inflammatory pathways, specifically Th17, may be contributing to the severe asthmatic phenotype seen in menopausal HDM-challenged mice [8]. Non-Th2 driven asthma, including Th1 and Th17, is often more severe and difficult to treat [31,32,33]. Other studies have demonstrated that Th1 and Th17 T cell differentiation is dependent on the conversion of glutamine to glutamate via the glutaminase enzyme [34,35]. The authors concluded that glutamine metabolism has a unique role in promoting Th17 cell differentiation but restricting Th1 and CTL effector cell differentiation. With the significant changes in “glutathione”, “alanine, aspartate, and glutamate”, and “d-glutamine and d-glutamate” metabolism observed in the serum of menopausal mice compared to control, it is possible that increased glutamic acid may be affecting the T-helper cell differentiation, which then influences the inflammatory response with allergen challenge. 

Pyroglutamic acid, an intermediate in glutathione metabolism that can be converted to glutamate by 5-oxoprolinase [36], is another metabolite upregulated in the serum of menopausal mice. A recent study detected significantly increased pyroglutamic acid, as well as glutamine, in the plasma of ovalbumin sensitized mice, which was then reduced with experimental treatment [37]. In addition, both metabolites were significantly associated with Penh values (measure of lung function) at various concentrations of methacholine challenge, eosinophil numbers, and Th17 cell percentages. Interestingly, pyroglutamic acid was not significantly upregulated in the non-menopausal HDM-challenged group compared to control in our study. Instead, we observed significant increases in the menopausal group compared to control and non-menopausal HDM challenged, as well as the menopausal HDM challenged compared to control, suggesting that menopause is the driving factor in the dysregulation of pyroglutamic acid in the VCD/HDM model. 

A previous study identified significantly impaired glutathione metabolism in the lung during an endoplasmic reticulum (ER) stress mouse model [38]. This same study also noted TMAO significantly downregulated in the ER stress model compared to control [38]. TMAO has also been identified in plasma and urine of asthma patients and is being considered as a possible biomarker for asthma exacerbation [39,40]. This is in line with our findings, in which we discovered significantly downregulated TMAO in the BALF of menopausal and HDM-challenged mice compared to control, and downregulated oxidized glutathione in serum of menopausal mice compared to control. Glutathione metabolism is believed to be involved in the regulation of redox homeostasis and relieving ER stress in the lung [38] and may a significant player in the pathogenesis of menopause-associated asthma. 

Histamine was significantly lower in the serum of HDM-challenged mice compared to control (Table 2) and had a significant negative correlation with airways hyper-responsiveness (Figure 5A). This contrasts with previous studies that demonstrated high histamine levels in the serum of sensitized animals as asthmatic patients [41]. This discrepancy may be due to the short half-life of histamine [42], or the timepoint at which collection occurred did not align with peak histamine release. Uridine, which is involved in nucleotide and pyrimidine metabolism, was significantly lower in the serum of menopausal mice compared to control, but significantly higher in menopausal HDM-challenged mice compared to menopausal mice (Table 2). Additionally, uridine had a significant correlation with airway hyper-responsiveness (Figure 5D). Previous literature has described uridine as being anti-inflammatory in models of lung inflammation [43]. 

We also identified several novel metabolites and metabolic pathways that have not been previously linked to asthma or lung disease, which warrant further investigation. Cytosine, which is one of the four base nucleotides that make up DNA, and cytidine, which is formed when cytosine is attached to a ribose ring in RNA, were both significantly upregulated in the BALF of menopausal HDM-challenged mice compared to all other groups, with the exception of menopausal HDM challenged vs. non-menopausal HDM challenged with cytosine (Figure 2C, Table 1). Both of these metabolites had significant positive correlations with airway hyper-responsiveness (Figure 5B,C). While not well studied, cytosine was elevated in patients with COVID-19 compared to healthy controls [44]. Additionally, researchers have shown that elevated cytosine levels are involved in the tryptophan/nicotinamide metabolic pathway, which has been linked to inflammatory signaling [45,46]. Coincidentally, nicotinuric acid was upregulated in serum of menopausal mice compared to control in our study. Cytidine was found to be upregulated in the sputum on patients with bronchial asthma [47,48]. 

9-octadecynoid acid, also known as stearolic acid, and acetamide were significantly elevated in the BALF of both HDM-challenged groups compared to controls, with the menopausal HDM-challenged mice having the highest concentrations (Figure 2A,B, Table 1). Acetamide had a significant positive correlation with airway hyper-responsiveness (Figure 5E). 9-Octadecynoic acid has only been described in a handful of metabolomics studies [49,50,51], none of which were related to the lung. Also very understudied, acetamide has been identified as a metabolomic biomarker for pregnancy complications [52,53,54,55]. With the significant differences in our studies and their strong associations with lung function, these two relatively understudied metabolites in regard to lung function and asthma serve as the most novel potential biomarkers for menopause-associated asthma and warrant additional study.

The major strength of this study lies in the VCD/HDM model of menopause in mice. This model results in robust and consistent changes in airway function in the menopausal HDM-challenged mice compared to non-menopausal mice, measures that typically yield high variability when using the Flexivent system. Additionally, we were able to assess metabolomic profiles from two compartments: the serum, which allowed us to understand the global effects of menopause and/or asthma on the animal, and BALF, which gave us a more precise view of the lungs and airways. The primary limitation of this study is the sample size. While n = 5 per group is sufficient to observe metabolomic changes, an increased sample size would aid in narrowing down the most significant metabolites and pathways that may be involved in disease severity and progression. Moreover, our samples were only collected at a single timepoint after the onset of menopause, so we are unable to infer longitudinal changes in metabolites across the menopausal transition. Additionally, a better understanding of the underlying changes in the central nervous system during menopause, related to hormonal fluctuation and the impact on lung function, remains a gap in knowledge that warrants further exploration in future studies.

In conclusion, our metabolomic analysis on samples from mice allowed us to better describe the metabolic landscape in the serum and BALF in the menopausal HDM-challenged mice in comparison to the other experimental groups. We discovered a large variety of significantly impacted metabolites and metabolic pathways in both compartments impacted by both menopause and asthma status. Additionally, PLS-DA analysis of the metabolites of the experimental groups was able to discriminate menopausal HDM-challenged and non-menopausal HDM-challenged groups. With this new information, we proposed possible mechanisms for disease, and potential novel biomarkers, which may be used to aid in the development of new therapies for a group of patients that currently have very few options. While additional studies are needed to validate results in humans and delve further into mechanisms of action driving asthma onset and reduced lung function in menopausal asthmatics, our study provides the foundation for detailing metabolomic changes in the lung and in circulation that may impact the pathological phenotypes manifested in menopause-associated asthma.

## Figures and Tables

**Table 1:** Significantly different individual metabolites in the BALF.

Comparison	Change	Metabolite	*p* Value	Fold Change
Menopause vs. Control	Downregulated	TMAO	0.007	0.11
		2-HydroxybenzoicAcid	0.012	0.30
		Threonine	0.014	0.59
		Homoserine	0.016	0.60
		Hippuric Acid	0.038	0.46
		Hydroxyproline	0.046	0.63
		5-Aminolevulinic Acid	0.049	0.67
	Upregulated	*4*-*Aminobutyric Acid*	*0.080*	*5.42*
HDM vs. Control	Downregulated	Fructose	0.041	0.60
		Ribose	0.045	0.61
		TMAO	0.048	0.25
	Upregulated	9-Octadecynoic Acid	0.001	3.31
		Acetamide	0.005	1.55
		Glycocyamine	0.012	2.15
Menopause HDM vs. Menopause	Upregulated	Cytosine	0.0003	3.96
		GlycoCyamine	0.005	1.92
		9-Octadecynoic Acid	0.011	2.24
		D-Ribose 5-phosphate	0.013	2.98
		Cytidine	0.023	2.21
		Phenylpyruvic Acid	0.040	1.86
Menopause HDM vs. HDM	Upregulated	Cytosine	0.00005	2.17
		Cytidine	0.015	2.05
		Glutamic Acid	0.033	2.44
		D-Ribose 5 Phosphate	0.047	1.96

Statistical significance acquired using *t*-test with a *p*-value cutoff of 0.05 and a fold change cutoff of 1.5. Italicized metabolites did not reach statistical significance of *p* ≤ 0.05 but were of particular interest.

**Table 2:** Significantly different individual metabolites in the serum.

Comparison	Change	Metabolite	*p* Value	Fold Change
Menopause vs. Control	Downregulated	2-Hydroxybenzoic Acid	0.007	0.12
		Oxidized Glutathione	0.007	0.61
		Mucic Acid	0.008	0.38
		5-aminolevulinic acid	0.008	0.67
		Acetylcholine	0.008	0.67
		Ribose	0.011	0.22
		Methyl Guanidine	0.011	0.34
		Xylose	0.011	0.19
		Hydroxyproline	0.012	0.65
		Carnosine	0.032	0.59
		Uridine	0.031	0.47
		GA3P	0.035	0.33
	Upregulated	Pyroglutamic Acid	0.0003	2.00
		Glucosamine	0.006	1.63
		3-hydroxyisovaleric Acid	0.007	1.63
		Myristic Acid	0.015	1.66
		Nicotinuric Acid	0.020	1.83
		4-Pyridoxic Acid	0.024	1.73
		Oxoglutaric Acid	0.032	1.82
		2-Methylglutaric Acid	0.037	1.77
		Indole 3-acetic Acid	0.042	2.33
HDM vs. Control	Downregulated	3-Methyladipic Acid	0.005	0.66
		Methyl-D-Mannopyranoside	0.023	0.61
		Histamine	0.026	0.40
		Carnosine	0.045	0.64
Menopause HDM vs. Menopause	Upregulated	Uridine	0.028	2.27
		Acetylcholine	0.034	1.66
Menopause HDM vs. HDM	Upregulated	Phosphocreatine	0.014	1.86

Statistical significance acquired using *t*-test with a *p*-value cutoff of 0.05 and a fold change cutoff of 1.5.

**Table 3:** Significantly impacted metabolic pathways in the BALF.

Comparison	Pathway Name	Match Status	*p* Value	Impact
Menopause vs. Control	*1. Arginine & Proline Metabolism*	*9/38*	*0.094*	*0.30*
	*2. Amino Sugar & Nucleotide Sugar Metabolism*	*1/37*	*0.100*	*0.0*
	*3. Phenylalanine Metabolism*	*3/12*	*0.110*	*0.62*
HDM vs. Control	1. Arginine & Proline Metabolism	9/38	0.007	0.30
	2. Amino Sugar & Nucleotide Sugar Metabolism	1/37	0.041	0.0
	3. Glycolysis/Gluconeogenesis	1/26	0.059	0.0
Menopause HDM vs. Menopause	1. Fatty Acid Elongation	1/39	0.003	0.0
	2. Fatty Acid Degradation	1/39	0.003	0.0
	3. Purine Metabolism	1/66	0.013	0.01
	4. Pyrimidine Metabolism	1/39	0.023	0.01
	5. Pentose Phosphate Pathway	3/22	0.024	0.17
	6. Biosynthesis of Unsaturated Fatty Acids	2/36	0.041	0.0
Menopause HDM vs. HDM	1. Pyrimidine Metabolism	1/39	0.015	0.01
	2. Pentose Phosphate Pathway	3/22	0.025	0.17
	3. Nitrogen Metabolism	1/6	0.033	0.0
	4. D-Glutamine & D-Glutamate metabolism	2/6	0.035	0.5
	5. Porphyrin and Chlorophyll Metabolism	3/30	0.035	0.03
	6. Arginine and Proline Metabolism	9/38	0.045	0.30
	7. Butanoate Metabolism	5/15	0.045	0.03
	8. Purine Metabolism	1/66	0.047	0.01

Numbers in front of pathway names correspond to numbers next to circles in Figure 3A–D. Statistical significance acquired using *t*-test with a *p*-value cutoff of 0.05. Italicized metabolic pathways did not reach statistical significance of *p* ≤ 0.05 but were of particular interest.

**Table 4:** Significantly impacted metabolic pathways in the serum.

Comparison	Pathway Name	Match Status	*p* Value	Impact
Menopause vs. Control	1. Glutathione Metabolism	7/28	0.0005	0.43
	2. Fatty Acid Elongation	1/39	0.004	0.0
	3. Fatty Acid Degradation	1/39	0.004	0.0
	4. Fatty Acid Biosynthesis	5/47	0.006	0.02
	5. Glycine, serine, & Threonine Metabolism	11/34	0.007	0.54
	6. Glycerophospholipid Metabolism	2/36	0.009	0.03
	7. Pentose & Glucuronate Interconversions	5/18	0.011	0.38
	8. Porphyrin and Chlorophyll Metabolism	3/30	0.014	0.03
	9. Pentose Phosphate Pathway	3/22	0.014	0.17
	10. Alanine, aspartate, & Glutamate Metabolism	9/28	0.019	0.59
	11. Vitamin B6 Metabolism	1/9	0.024	0.0
	12. Butanoate Metabolism	5/15	0.033	0.0
	13. D-Glutamine & D-Glutamate metabolism	3/6	0.036	0.5
	14. Beta-Alanine Metabolism	3/21	0.038	0.06
	15. Pyrimidine Metabolism	5/39	0.038	0.09
	16. Tryptophan Metabolism	4/41	0.050	0.25
HDM vs. Control	1. Glycerophospholipid Metabolism	2/36	0.023	0.03
	2. Selenocompound Metabolism	1/20	0.024	0.0
	3. Histidine Metabolism	9/16	0.026	0.71
	4. Glycine, Serine, & Threonine Metabolism	11/34	0.041	0.54
Menopause HDM vs. Menopause	1. Glycerophospholipid metabolism	2/36	0.033	0.03
	2. Arachidonic Acid Metabolism	1/36	0.035	0.0
	3. Pyrimidine Metabolism	5/39	0.042	0.09
	4. Selenocompound Metabolism	1/20	0.045	0.0
Menopause HDM vs. HDM	1. Arginine and Proline Metabolism	8/38	0.015	0.38

Numbers in front of pathway names correspond to numbers next to circles in Figure 3E–H. Statistical significance acquired using *t*-test with a *p*-value cutoff of 0.05.

## Data Availability

The data presented in this study are available on request from the corresponding author. Ongoing studies with this particular data set are still in progress.
